# COVID-19 and Related Vaccinations in Children: Pathogenic Aspects of Oral Lesions

**DOI:** 10.3390/children10050809

**Published:** 2023-04-29

**Authors:** Federica Di Spirito, Francesco D’Ambrosio, Maria Pia Di Palo, Francesco Giordano, Nicoletta Coppola, Maria Contaldo

**Affiliations:** 1 Department of Medicine, Surgery and Dentistry, University of Salerno, 84084 Salerno, Italy; fradambrosio@unisa.it (F.D.); mariapia140497@gmail.com (M.P.D.P.); frgiordano@unisa.it (F.G.); nicolettacoppola95@gmail.com (N.C.); 2 Multidisciplinary Department of Medical-Surgical and Odontostomatological Specialities, University of Campania “Luigi Vanvitelli”, 80138 Naples, Italy

**Keywords:** COVID-19, SARS-CoV-2, oral manifestations, vaccination

## Abstract

Various clinical manifestations of SARS-CoV-2 infections and adverse reactions to COVID-19 vaccination have been described in children. The present narrative review aimed to collect and synthesize reported findings on oral lesions detected in SARS-CoV-2-positive subjects following COVID-19 EMA-authorized and WHO Emergency Use Listing-approved vaccine administration in the pediatric population to detail their clinical features and highlight possible pathogenic aspects of those lesions based on current evidence. Few and incomplete reports were retrieved from the literature, probably because most lesions belonged to a broad spectrum of systemic diseases and syndromes and were nonspecific or inaccurately described. The most common oral lesions in pediatric SARS-CoV-2-positive patients were erosive–ulcerative lesions and macules/petechiae, primarily erythematous. In the context of COVID-19 vaccination, oral adverse reactions were rare and typically presented as erosive–ulcerative lesions, with EM-like or unspecified patterns. Future studies should investigate oral lesions in SARS-CoV-2-positive subjects and after COVID-19 vaccination in the pediatric population, taking into account viral variants and newly developed vaccines. Deeper insight into oral lesions detectable in pediatric SARS-CoV-2-positive subjects and after COVID-19 vaccination may increase clinicians’ ability to improve multidisciplinary pediatric oral and general care.

## 1. Introduction

Coronavirus disease 2019 (COVID-19), caused by Severe Acute Respiratory Syndrome Coronavirus 2 (SARS-CoV-2), most commonly presents with symptoms such as fever, asthenia, dry cough, headache, dyspnea, vomiting, diarrhea, abdominal pain, hyposmia, dysgeusia, and oropharyngeal involvement [1]. While at the beginning of the COVID-19 pandemic the disease was rarely reported in children and generally described as asymptomatic or mild [2], pediatric cases later increased, and some presented with a particular form of a new multi-systemic syndrome likely associated with SARS-CoV-2 infection that also affects the oral cavity [2,3]. Indeed, contrary to SARS-CoV-1 (Severe Acute Respiratory Syndrome Coronavirus 1), SARS-CoV-2 has been related to several oral manifestations, including oral mucosa lesions, in both adults and young people [1,2,3,4].

Moreover, COVID-19 vaccination, effective in preventing severe forms of COVID-19 and reducing the risk of transmitting the virus and becoming infected [5,6], has also been recommended for the pediatric population [7]. Albeit rare, adverse drug reactions (ADRs) to COVID-19 vaccines have been reported in both pediatric [3] and adult subjects [5,6], including those involving the oral cavity.

Therefore, the present review aimed to collect and synthesize narratively reported findings on oral lesions detected in SARS-CoV-2-positive subjects and after the administration of the COVID-19 European Medicines Agency (EMA)-authorized and World Health Organization (WHO) Emergency Use Listing-approved vaccines in the pediatric (≤18 years of age) population to describe their clinical features and highlight the possible pathogenic aspects of oral lesions based on the current evidence.

## 2. Materials and Methods

The authors searched the PubMed, Web of Science, Scopus, and Google Scholar databases through 30 December 2022 for records reporting oral mucosa lesions in the pediatric (≤18 years old) population among SARS-CoV-2-positive cases and after the administration of the COVID-19 EMA-authorized [8] and WHO Emergency Use Listing-approved vaccines [9] and describing the possible underlying pathogenic mechanisms.

The following keywords (free-search terms) were used: 

COVID-19 OR coronavirus disease 2019 OR SARS-CoV-2 OR novel coronavirus OR anti-SARS-CoV-2

AND

oral manifestations OR oral lesions OR mucosal lesions OR

AND

Positive OR vaccination OR vaccine OR vaccines.

References were collected and managed through the Mendeley Reference Manager software. 

Any study published without a date restriction describing oral lesions and potentially related pathogenic mechanisms was included. Records that did not report oral lesions in the pediatric (≤18 years old) population among SARS-CoV-2-positive cases or after at least one administration of the COVID-19 EMA-authorized [8] and WHO Emergency Use Listing-approved vaccines [9] and studies involving adult (<18 years old) subjects only were excluded.

## 3. Results

A total of 6863 records were initially retrieved from Google Scholar, as well as 226 from PubMed, 168 from Scopus, and 98 from Web of Science. After duplicate elimination, those records not compliant with the eligibility criteria were excluded. Ten studies [3,10,11,12,13,14,15,16,17,18] exclusively involved SARS-CoV-2-positive pediatric (<18 years old) cases with oral lesions and/or pediatric subjects with oral lesions after at least one administration of the COVID-19 EMA-authorized [8] and WHO Emergency Use Listing-approved vaccines [9].

### 3.1. SARS-CoV-2 Infection in Pediatric Subjects

At the beginning of the COVID-19 outbreak, children appeared to be less susceptible to SARS-CoV-2 infection than adults or more inclined to develop the asymptomatic and mild forms of the disease, which may have led to an underestimation of epidemiologic data in young patients [10]. Age-related differences in viral receptor expression, immune system competence, and comorbidity rates partially explained the lower prevalence of COVID-19 infection in pediatric patients [11]. 

However, especially after the spread of the Omicron virus variant and because of the relaxation of social and health measures, the number of young people infected with SARS-CoV-2 increased [9], and a hyperinflammatory syndrome likely related to SARS-CoV-2 infection called “Multisystem Inflammatory Syndrome in Children” (MIS -C) or “Pediatric Inflammatory Multisystem Syndrome Temporally Associated with COVID-19” (PIMS-TS) [10,11] was observed. This syndrome affects the oral cavity like other known diseases, such as Kawasaki disease (KD), Macrophage Activation Syndrome (MAS), Toxic Shock Syndrome (TSS), and Secondary Hemophagocytic Lymphohistiocytosis (SHLH) [10,11]. 

According to UNICEF data covering 105 countries from the start of the COVID-19 pandemic to 1 January 2023, 8.4% of children aged 0–4 years, 4.6% aged 5–9 years, 5.8% aged 10–14 years, and 6–7% aged 15–19 years have been infected with SARS-CoV-2 [19]. 

Among pediatric SARS-CoV-2-positive subjects, the risk of severe COVID-19 disease, with possible hospitalization and death, was expected to increase when comorbidities occurred, although pediatric patients hospitalized for COVID-19 usually did not have multiple diseases [11]. Nevertheless, Peckham et al., evaluating 3,111,714 pediatric cases worldwide, revealed that male and female children had the same infection risk, but males had a higher risk of hospitalization and death [20].

#### 3.1.1. Oral Lesions in Pediatric SARS-CoV-2-Positive Subjects

Very heterogeneous terms for primary oral lesions have been found in the literature, probably due to the fact that oral lesions have been reported mainly in the context of systemic syndromes and thus encountered and described by healthcare providers not specialized in oral medicine [21], with the additional risk of underestimation, as well as overestimation, of some primary oral lesions, as well as misdiagnosis [1].

Oral lesions were frequently described in association with skin lesions, and their overall prevalence was estimated to similarly range between 2% and 20% of pediatric SARS-CoV-2-positive subjects [22]. A systematic review by Erbas et al. [23] found that 60% of subjects with oral lesions also had skin involvement, and the association between oral mucosal and skin manifestations was greater in the group with oral lesions attributable to KD, which is more common in the pediatric population.

The reported prevalence of oral lesions in pediatric SARS-CoV-2-positive subjects may have been underestimated because most subjects were not diagnosed with COVID-19 because they were a-/oligosymptomatic [10,11] or because they were in in-home care and did not undergo oral examinations.

Furthermore, pediatric cases with severe COVID-19 have been described as having a higher incidence of oral lesions. Accordingly, similar to adult SARS-CoV-2-positive subjects (M:F = 1.78:1), oral lesions were more commonly described in young males compared with females (M:F = 1.33:1), who were also more frequently hospitalized [1]. It is very likely that the higher hospitalization rate of male pediatric patients led to the earlier diagnosis of oral lesions since it may be more difficult to detect oral lesions in patients in home care. However, based on the retrieved data, oral lesions observed in pediatric subjects with COVID-19 are likely to be found in males with severe forms of the illness, as previously proposed by Orilisi et al. [24] for adult males with severe COVID-19, although no studies have been conducted on young patients. 

From this perspective, oral lesions may represent a sign of disease exacerbation [21,25] resulting from systemic involvement and, thus, indirectly suggest adjusting the therapeutic approach to physicians. 

However, it should be noted that oral lesions have also been proposed as prodromic signs in pre-/asymptomatic SARS-CoV-2 infections, similar to smell and taste [21,25]. Indeed, Pasquini Neto et al. suggested that both oral and dermatologic manifestations may be initial signs of SARS-CoV-2 infection to watch for, as infected individuals may not show respiratory symptoms until 14 days after infection [26]. The recognition of these lesions is especially important in the pediatric population, which usually presents with mild to moderate forms of the disease [12].

Two systematic reviews specifically examined oral lesions in younger (<18 years old) SARS-CoV-2-positive subjects [3,13]. 

In the first, the following primary oral manifestations were reported in 35 pediatric subjects, categorized according to a previous classification of primary oral lesions [1]: maculae and petechiae (11.63%), in the form of erythema (4.65%), purpura (2.33%), ecchymosis (2.33%), and petechiae (2.33%); nonspecific ulcers and erosions (6.98%); other oral lesions (81.4%), especially presenting as hyperemic pharynx (23.26%); oral candidiasis (11.63%); fissures (4.65%); coated tongue (4.65%); hypertrophic papillae (2.33%); geographic tongue (2.33%); crusts (2.33%); swollen (2.33%); and nonspecific oral mucosal changes (27.91%) [3]. 

The second systematic literature review examined 624 pediatric cases diagnosed with MIS-C or KD associated with SARS-CoV-2 infection [13]. Oral manifestations were one of the most common signs of MIS-C and were not different from the oral lesions reported in KD cases [13]. In both diseases, changes in the oral cavity were noted as one of the earliest symptoms, and erythema of the oral mucosa and tongue was reported, accompanied by aphthous/ulcerative lesions in some cases [13]. The lips were affected by erythema, edema, dryness, or superficial scaling [13]. Burning or pain that prevented or limited normal food intake was rarely reported [13]. 

Despite the different terminology used in the two systematic reviews [3,13], macules/petechiae, predominantly in erythematous forms, and ulcerative/erosive lesions appeared to be the most common oral lesions in pediatric SARS-CoV-2-positive subjects, whether or not they developed SARS-CoV-2-associated syndromes.

Macular lesions were also the predominant skin manifestations observed in SARS-CoV-2-positive children, followed by erosions [12,14,26,27]. In agreement, Shah et al. [14] showed that most pediatric subjects had erythematous maculopapular skin eruptions, which are often the typical dermatological manifestation of viral infections, although their distribution was not typically cephalocaudal as in other viral exanthemas. Indeed, in SARS-CoV-2-positive children, the sites typically affected were the acral regions of the hands and feet [14]. In addition, while maculopapular viral exanthemas generally heal with exfoliation, the lesions described in SARS-CoV-2-positive children typically progressed to erosions, vesiculation, and crusting [14]. Consequently, acral distribution and distal extremities with the appearance of exanthema are more likely attributable to Parvovirus B19 infections, causing the so-called “glove and sock syndrome” and “acropetecic syndrome” [27].

Nonetheless, the distribution of maculopapular lesions on the distal extremities and the possible association with oral lesions are commonly observed in EM, an immune-mediated reaction caused by infections or drugs [27], which could be, in turn, considered indicative of SARS-CoV-2 infection.

Oral erosions and ulcerations were even more common in SARS-CoV-2-positive adults than in the pediatric population. In fact, erosive–ulcerative oral lesions were the most common and accounted for almost half of the primary oral lesions (48.96%) in the adult population, followed by maculopapular (16.44%) and vesiculobullous (4.97%) lesions and white plaques (0.25%) [1]. Other manifestations not classified as primary lesions of the oral cavity were reported to a lesser extent in adults (20.64%) in the form of not-defined alterations of the oral mucosa (27.91%), hyperemia of the pharynx (23.26%), candidiasis (11.63%), and desquamative gingivitis (0.90%) [1]. Two cases of necrotizing periodontitis are reported in the literature [15,28], both in adult patients. To the best of our current knowledge, no cases of necrotizing periodontitis have been found in pediatric patients. Necrotizing periodontal lesions may be favored by coinfection between periodontal pathogens and SARS-CoV-2, which is able to overcome the epithelium of periodontal pockets via the viral ACE-2 receptors expressed there [22]. The same virus is then able to alter the function of sulcular epithelial cells in individuals with severe COVID-19 and induce a state of immunological derangement [22]. However, the reason why these manifestations were found only in adult individuals remains unknown.

#### 3.1.2. Possible Pathogenic Aspects of Oral Lesions in Pediatric SARS-CoV-2-Positive Subjects

The main potentially pathogenic mechanisms responsible for oral lesions following SARS-CoV-2 infection are related to the direct viral cytopathic effect, immune–inflammatory dysregulation, and COVID-19 treatments (pharmacological or with mechanical ventilation) [29,30,31,32].

A SARS-CoV-2 infection can induce a direct viral cytopathic effect [33]. The virus is able to enter oral epithelial cells via angiotensin-converting enzyme 2 (ACE2) receptors [33], which are the route of entry of the virus into the respiratory epithelium and are also widely expressed in oral epithelial cells, particularly those of the tongue and salivary glands [34].

Histological findings from the oral lesions of SARS-CoV-2-infected subjects showed the presence of exocytosis and vacuolization (100%) in the epithelial layer and inflammatory infiltrate (60%), microvascular thrombosis (60%), and hemorrhage (40%) in the mesenchymal later [35]. Thus, the virus, penetrating into the epithelial cells, induces an increase in cell permeability [33] and the formation of vesicles and blisters that are difficult to detect in the oral cavity due to masticatory forces, resulting in erosive–ulcerative lesions. 

Moreover, SARS-CoV-2 is also able to cause direct damage to endothelial cells as a result of internalization via the ACE2 receptor of the cleaved spike protein, which is capable of causing the vasoconstriction and endotheliopathy observed in COVID-19 [30]. 

In the nucleus of endothelial cells, intermingled with thrombotic tissue, caspase-3 has been found, demonstrating the activation of cellular apoptotic processes [36]. Thromboses in capillaries and small arteries are common histological findings, and fibrinoid necrosis of vessels was found in many cases [36]. SARS-CoV-2-directed endothelial damage and prothrombotic states may generate focal areas of variable degrees of ischemia, resulting in ulcers on the oral mucosa and livedo racemose or retiform purpura or acral ischemia on the skin [30]. 

The authors suggest that direct cytopathic damage may be less common in children than in adults since the role of ACE-2 receptors is essential, and whose gene is less expressed in young subjects who also have milder pulmonary manifestations than adults [11,37].

Oral lesions may also be an epiphenomenon of immune–inflammatory hyper-reactions; in fact, SARS-CoV-2 infection induces cytokine storms; neutrophil chemotaxis in the inflammatory sites of the oral mucosa [38]; and the dysregulation of pro-inflammatory T helper cells, which produce interleukin-17 (IL-17) [32]. In particular, MIC-S is characterized by high levels of inflammatory cytokines such as IL-1β, IL-6, IL-8, IL-10, IL-17, and interferon-γ (INF-γ) [39]. 

Interferons play a crucial role in the immunological response against viruses [30] but are also associated with the pathogenesis of skin manifestations such as chilblains [30]. The cytokine storm of COVID-19 [33] and the strong inflammatory response mediated by the IFN in children, on the one hand, inhibit viral replication, but on the other, they are associated with the appearance of mucocutaneous lesions [30]. 

Similar to the type of skin lesions in chilblains [30], the authors suggest that the inflammatory vascular damage manifests itself in the form of maculae and petechiae (as erythema, purpura, ecchymosis, and petechiae) in the oral cavity. 

Manifestations of immune–inflammatory hyper-reactivity of the skin (chilblains) and oral mucosa (maculae and petechiae) have been reported more frequently in children than the corresponding manifestations of direct viral cytopathic damage (livedo racemosa and retiform purpura or acral ischemia for the skin and ulcers for oral mucosa) [3,30,40].

Other oral manifestations, such as map tongue, gingival, and pharynx hypertrophy, could be the manifestation of acute inflammatory disease, especially in predisposed children with a family or personal history of atopic diathesis [41]. In fact, map tongue is more common in patients with a tendency to develop acute inflammatory diseases on the surfaces of contact with the external environment [41]. 

Allergic skin manifestations have also been reported in SARS-CoV-2-positive subjects by Farinazzo et al. [30] in the form of urticaria. The authors point out that, in some cases, urticaria was not attributable to the use of drugs for the treatment of COVID-19, as their interruption did not induce the regression of urticaria [30]. It is, therefore, hypothesized that urticaria may be associated, in some cases, with drugs, while in others, it may be directly associated with viral infection [30]. 

Another possible pathogenetic mechanism that could be linked to the immune–inflammatory hypo-reaction of hosts with COVID-19 is one that favors the colonization of *Candida albicans*, a normal commensal of oral microbiota [42]. Opportunistic infections by *Candida albicans* are known to be associated with cognitive impairment, multiple comorbidities, poor oral hygiene, and the prolonged use of antibiotics and steroids, which are drugs used to treat COVID-19 [32]. 

A decreased focus on oral hygiene practices was reported among adolescents during the lockdown period [43]. 

In addition, SARS-CoV-2-positive subjects may suffer from xerostomia because of the direct cytopathic mechanism of the ductal epithelial cells of the salivary glands [32] expressing high levels of ACE2 [34]. 

The dental biofilm promotes the adhesion of *Candida albicans* and reduces the production of antimicrobial proteins such as lysozyme and lactoferrin, reducing the antimicrobial properties of saliva and predisposing the subject to oral candidiasis [42,44].

The oral manifestations of the herpetiform type reported in children [40] may be attributable to direct immune reactions to SARS-CoV-2 and a state of inflammatory–immune hypo-reaction. 

Mucocutaneous manifestations have been reported to be attributable to reactivations of latent herpes simplex-1 (HSV-1) or varicella zoster infections [31,45]. HSV-1 is able to establish a latent infection in the neuronal cells of the ganglia due to the presence of CD8+ T cells around them [45]. Lymphopenia is often associated with SARS-CoV-2 infections [31], so immune hypo-dysregulation caused by COVID-19 and T lymphocytes polarization toward the spike protein [29] involves the assembly of viral components and the clinical reactivation of the infection, similar to what happens in case of stress or hormonal changes [45]. The affected area of reactivated HSV-1 manifestation is considered a locus for minoris resistentiae and an immunocompromised district. SARS-CoV-2 induces an immuno-inflammatory response leading to the reactivation of HSV-1 by mimicking an event similar to the Koebner phenomenon, which has been called the “Wolf isotopic response” [30].

Adults are subject to a natural immunosenescence [46], which causes further depletion of the T lymphocytes that guard the ganglia where latent HSV-1 resides. Therefore, it is possible to hypothesize that the aforementioned pathogenetic mechanism is more likely among adults.

The Centre for Disease Control COVID-19 Response Team has estimated that 20% of SARS-CoV-2-positive pediatric patients were hospitalized, compared with 33% of subjects between 18 to 64 years old [16,47]. Other studies have reported that 2.1% of hospitalized children needed intubation [16,47].

It has been suggested that oral ulcerative lesions may be caused by medical intubation and mechanical ventilation devices, especially when patients with severe COVID-19 are placed prone [24,48]. Mechanical trauma after orotracheal tube friction and compression can cause ulcerations, edema, and hematomas of the lips [24,48]. 

Furthermore, intubated patients are unable to carry out normal oral hygiene practices, and this favors, in addition to the state of immunodepression caused by COVID-19, *Candida albicans* infections.

Oral lesions in SARS-CoV-2-positive subjects may be adverse reactions to drugs administered for the treatment of COVID-19. In fact, the inflammatory cytokine cascade may trigger hypersensitive reactions to drugs [30].

Diagnoses of Erythema Multiforme (EM) and Steven Johnson Syndrome (SJS) have been reported in pediatric subjects [40], which are acute hypersensitivity reactions often associated with infections or drugs [5]. 

According to a recent systematic review by Panda et al. [49], drugs that are commonly used to treat COVID-19 in children include antivirals (such as remdesivir, ritonavir, and lopinavir), anti-inflammatory drugs (corticosteroids, acetylsalicylic acid, and indomethacin), hydrochloroquinolines, antibiotics, anthelmintics, intravenous immunoglobulins, interferon, monoclonal antibodies (such as tocilizumab and infliximab), anticoagulants (heparin and low-molecular-weight heparin), vitamins C and D, and zinc. Some of these drugs, especially hydroxychloroquine, when used in the treatment of COVID-19, may induce SJS, EM, or EM-like eruptions in pediatric patients [50,51]. The prolongated use of high doses of antibiotics and corticosteroids can result in immunosuppression, which favors opportunistic microorganisms such as *Candida albicans* [5,42]. 

**Figure 1:**
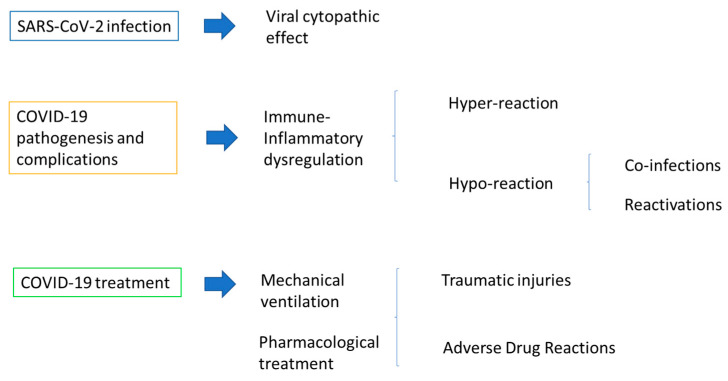
Pathogenic mechanisms potentially underlying oral lesions detected in pediatric SARS-CoV-2-positive subjects.

Figure 1 illustrates the pathogenic mechanisms potentially underlying oral lesions detected in SARS-CoV-2-positive subjects.

### 3.2. COVID-19 Vaccination in Pediatric Subjects

Pediatric patients can be protected from SARS-CoV-2 infection, especially from complications after COVID-19 and MIS -C/PIMS-TS, by vaccination [11]. 

Considering the risk–benefit ratio, vaccination is recommended for 12- to 17-year-olds in all European countries by the European Center for Disease Prevention and Control despite the possibility of side effects. Some countries strongly recommend booster vaccinations for the same age group [11].

The mRNA vaccines BNT162b2, Comirnaty (Pfizer-BioNTech), and mRNA-1273, Spikevax (Moderna), have been approved for children aged 6–11 years old by the European Medicines Agency (EMA) [3]. The FDA has approved the use of Spikevax, mRNA-1273 (Moderna), in children younger than 5 years of age [3].

According to the Centers for Disease Control and Prevention, 17.8 million (68%) of U.S. children aged 12–17 years have received at least one vaccine dose, as have 11.1 million (39%) aged 5–11 years and 2.0 million (12%) aged 6 months–4 years (by 1 February 2023) [52].

The administration of drugs [53,54] and vaccines [6,55,56] can lead to adverse drug reactions. The main adverse reactions after COVID-19 vaccination include local pain, swelling, or redness at the injection site and symptoms resembling COVID-19 illness, such as general weakness, headache, chills, fever, joint and muscle pain, nausea, and diarrhea [57]. 

Although rare, mucocutaneous side effects affecting the orofacial area and oral mucosa have been previously described after vaccination against influenza, hepatitis B, Mumps–Measles–Rubella, Clostridium tetani, and others [10,11,55], similar to those following the COVID-19 vaccine. Specifically, mucocutaneous adverse reactions to COVID-19 vaccines include local reactions at the injection site, delayed local reactions in the form of morbilliform/erythematous/maculopapular/vesicular/vesiculopapulareruptions, urticaria, EM inflammatory reactions to dermal fillers, erythromelalgia, lichen planus, the reactivation of varicella zoster or herpes simplex infections, pityriasis rosea or rosea-like lesions, petechiae, pernicious purpura, and chilblains [57,58,59,60]. 

#### 3.2.1. Oral Lesions in Pediatric Subjects following COVID-19 Vaccination 

Systemic and mucocutaneous side effects affecting the orofacial area and oral mucosa after vaccination have generally been more frequent in adults, whereas few postvaccine adverse events have generally been recorded in children. For example, the Vaccine Adverse Event Reporting System recorded more than 1000 cases of mucosal and/or skin reactivation of herpes zoster infections, mainly affecting persons older than 60 years [58]. In the same vein, few cases of ADR were reported in young subjects despite the immunogenicity of vaccines being reduced in the elderly due to immunosenescence. In young males, myocarditis/pericarditis has rarely been described after a few days following COVID-19 vaccination [11,61]. The occurrence of myocarditis/pericarditis is considered a rare complication compared with the possibility of contracting a SARS-CoV-2 infection [11]. 

Oral ADRs associated with COVID-19 are rare complications, usually described along with mucocutaneous manifestations. Among orofacial ADRs, tongue, throat, and face swelling; Bell’s facial paralysis; and heterogeneous mucocutaneous lesions, including oral mucosal lesions in adults [62], have been noticed following the administration of COVID-19 vaccines.

The finding of a very low prevalence of oral lesions after COVID-19 vaccination may have been biased because outpatient epidemiologic data on oral ADRs in the pediatric population provided little information on their frequency, presentation, and causative medications [63]. Moreover, oral lesions in children who have received at least one dose of the COVID-19 vaccine may have been undiagnosed before their spontaneous recovery or misdiagnosed with other common clinical manifestations, such as those related to *Candida albicans* or herpes simplex virus type 1 infections, which do not require special diagnostic testing in routine clinical practice [3]. Conversely, more attention has been cautiously paid to severe adverse effects with systemic involvement in pediatric patients after COVID-19 vaccinations [3].

The primary oral lesions identified were erosions and ulcers (40%) with herpetiform and nonspecific patterns and maculae and petechiae (20%); other lesions (40%) were evenly described as crusts and pseudomembranous lesions [3]. The higher prevalence of erosive–ulcerative lesions observed may be explained by the fact that parents of young patients may only rarely detect oral manifestations, especially if they are asymptomatic. However, oral erosions and ulcers, which are usually symptomatic, are considered the most common oral ADRs caused by COVID-19 vaccines, and pseudomembranes and crusts, representing their natural progression, have been reported in 40% of cases [3]. 

The cases described were finally diagnosed with Behçet’s disease [61] and EM [64], which was also frequently diagnosed in pediatric SARS-CoV-2-positive subjects. EM mainly occurs in patients with a history of herpes simplex virus type 1 or Mycoplasma pneumoniae infections, which are more common in adolescents and young adults [65].

Oral erosive–ulcerative lesions (34.5%), with unspecified (20.7%) or EM-like patterns (13.8%), were also the most common primary oral lesions in adult subjects with at least one dose of the COVID-19 vaccine [56], followed by white plaques (10.3%), vesiculae and bullae (6.9%), and erythematous macules (6.9%); no specific features of other lesions were reported (34.5%).

Conversely, white lesions seem to have a greater predilection for adulthood. Of note, oral lichenoid reactions and oral lichen planus (OLP) have been reported in adults [54,66,67,68], followed by SJS and EM [64,69,70], although no cases of lichenoid reactions and oral lichen planus (OLP) have been recorded in children [40]. OLP occurs preferentially in adults, likely because of a lower incidence of autoimmune diseases, stress levels, and disease awareness in children [40,71] Indeed, pediatric LP has an approximate prevalence of 0.03% compared with 1–2% among adults.

However, manifestations of LP have been documented in children after hepatitis B virus vaccinations [71,72], probably following a cell-mediated autoimmune response triggered by the viral S epitope [72]. Therefore, it seems relevant to pay more attention to the diagnosis of OLP, also among the oral ADRs, in the pediatric population. Pediatric OLP typically presents as reticular [71] and needs to be differentiated from lichenoid lesions and reactions, discoid lupus erythematosus, graft-versus-host disease, leukoplakia, pseudomembranous candidiasis, and frictional keratoses [72].

#### 3.2.2. Possible Pathogenic Aspects for Oral Lesions following COVID-19 Vaccination in Pediatric Subjects

The pathogenetic mechanisms potentially underlying oral lesions from the COVID-19 vaccine are, in part, similar to those related to viral infection and include hypersensitivity reactions (from molecular mimicry, cross-reactivity, and autoimmunity), the reactivation of latent viral infections, and allergies to vaccine excipients [5,29].

Hypersensitivity reactions typically occurred with a lichenoid pattern or were EM-like [29]; however, as previously mentioned, no cases of lichenoid reactions in children have been reported. 

Hypersensitivity reactions are the consequence of the direct immune response to the lipid nanoparticles of mRNA vaccines or the apoptosis of oral epitheliolytic-expressing vaccine antigens and are targeted by cytotoxic CD8+ T lymphocytes [5,29]. Lymphocytes T and B are activated following BNT162b2 administration, and IL-2, IL-4, IL-17, and IL-21 are associated with autoimmune diseases, especially in genetically susceptible individuals [5]. mRNA vaccines might also trigger myeloid or plasmacytoid dendritic cells responsible for hypersensitivity or autoimmunity reactions [5]. 

It has also been hypothesized that autoimmune reactions to the vaccine are due to molecular mimicry and cross-reactivity mechanisms [29]. Anti-SARS-CoV-2 antibodies would react with unknown aminoacidic sequences of oral epitheliolytic glycoproteins that have sequences similar to those of the virus [29]. 

Alternatively, the vaccine spike protein could enter host cells through the ACE2 receptor and induce a direct cytopathic mechanism as described above for SARS-CoV-2 infections. 

Varicella zoster and herpes simplex virus reactivations have been reported after yellow fever, hepatitis A, rabies, and influenza vaccines [7]. Furthermore, the COVID-19 vaccine may induce HSV reactivation [5,7,29] because the mRNA-based vaccine allows for the transcription of the viral spike protein [29], which triggers the same mechanisms described for SARS-CoV-2 infection.

The last pathogenetic mechanism is related to an allergy to vaccine excipients. In particular, cases of delayed mucocutaneous reactions (>24 h) have been reported for allergies to polysorbate 80, which is contained in mRNA-based vaccines to prevent the rapid degradation of the molecule [5,29]. Another allergizing excipient is polyethylene glycol, added to vaccines to increase particle stability and immunogenic capacity [5]. 

**Figure 2:**
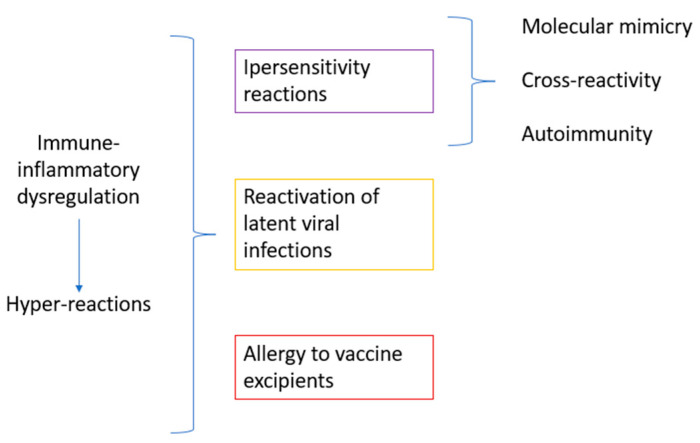
Pathogenetic mechanisms potentially underlying oral lesions following COVID-19 vaccination in pediatric subjects.

Figure 2 summarizes the potential pathogenetic mechanisms of oral lesions following COVID-19 vaccination in pediatric subjects.

## 4. Discussion 

The present narrative review aimed to collect and summarize the reported findings of oral lesions detected in SARS-CoV-2-positive subjects and after the administration of the COVID-19 EMA-authorized and WHO Emergency Use Listing-approved vaccines in the pediatric (≤18 years of age) population to describe their clinical features and to highlight possible pathogenetic aspects of these lesions based on current evidence. 

The prevalence of oral lesions in pediatric SARS-CoV-2-positive subjects may have been underestimated due to a lack of COVID-19 diagnosis and in-home care without oral examination [10,11]. Moreover, the inconsistent terminology used to describe primary oral lesions in the literature poses a risk of both underestimation and overestimation, as well as misdiagnosis [1,21]. 

Oral lesions were observed alongside skin lesions in 60% of cases, particularly in those with oral lesions linked to Kawasaki disease, which is more prevalent in the pediatric population, with a similar prevalence of 2% to 20% in pediatric SARS-CoV-2-positive subjects [16]. A systematic review by Erbas et al. [23] found that oral and skin manifestations were associated with this phenomenon. Oral lesions may indicate disease exacerbation [21,25], which could help physicians adjust their therapeutic approach accordingly. Interestingly, oral lesions have also been suggested as prodromic signs in pre-/asymptomatic SARS-CoV-2 infections, similar to symptoms involving smell and taste [21,25]. 

Most oral lesions reported in pediatric SARS-CoV-2-positive subjects were part of the multisystem syndromic spectrum; hence, they were not described in detail or not described by oral medicine physicians with heterogeneous denominations. Indeed, oral lesions are one of the earliest symptoms of MIS-C and KD [10,11,12,13,14,15,16,17,18,19,20,21,22,23,24,25,26,27].

Despite the heterogeneous terminology, ulcerative/erosive phenotype lesions and macules/petechiae, predominantly in an erythematous form, appear to be the most common oral lesions in pediatric SARS-CoV-2-positive patients [10,11,12,13,14,15,16,17,18,19,20,21,22,23,24,25,26,27].

The main potential pathogenic mechanisms responsible for such oral lesions after SARS-CoV-2 infection may be the following: the direct cytopathic effect of the virus entering epithelial or endothelial cells, increasing cell permeability and resulting in direct cellular damage; immune–inflammatory dysregulation (immune–inflammatory hyper-reactions due to COVID-19-induced cytokine storms, especially in MIC-S); host immune–inflammatory hypo-reactions that favor opportunistic infections; COVID-19 pharmacological therapies, possibly causing immunosuppression; or mechanical ventilation, possibly causing friction and compression of the mucosa [27,28,29,30,31,32,33,34,35,36,37,38,39,40,41,42,43,44,45,46,47,48].

Previous reports have described mucocutaneous side effects affecting the orofacial area and oral mucosa following vaccination against various diseases, such as influenza, hepatitis B, Mumps–Measles–Rubella, Clostridium tetani, and others, including COVID-19 [10,11,53]. These adverse events can be local, delayed, or systemic and take many forms, including eruptions, urticaria, and inflammatory reactions. While oral adverse drug reactions (ADRs) associated with COVID-19 vaccination are rare, they have been reported in adults, including tongue, throat, and face swelling; Bell’s facial paralysis; and various mucocutaneous lesions [59]. In children, the certain prevalence of oral lesions is unknown due to insufficient epidemiologic data and the possible misdiagnosis of oral lesions as other common clinical manifestations [22]. Adverse oral reactions to COVID-19 vaccines were extremely rare and occurred mainly as erosive–ulcerative lesions with EM-like or unspecified patterns [58]. In contrast, white lesions are more commonly seen in adults. Oral lichenoid reactions and oral lichen planus (OLP) have been reported in adults [52,64,65,66], followed by SJS and EM [61,66,67]. However, no cases of lichenoid reactions and OLP have been recorded in children [3], likely because of a lower incidence of autoimmune diseases, stress levels, and disease awareness. Nevertheless, pediatric LP can still occur, as evidenced by cases following hepatitis B virus vaccination [68,69]. As such, it is important to differentiate pediatric OLP from lichenoid lesions and reactions, discoid lupus erythematosus, graft-versus-host disease, leukoplakia, pseudomembranous candidiasis, and frictional keratoses [69] among the oral ADRs.

The pathogenetic mechanisms potentially underlying oral lesions due to COVID-19 vaccination may include hypersensitivity reactions due to molecular mimicry, cross-reactivity, autoimmunity, the reactivation of latent viral infections, and allergies to vaccine excipients, such as polysorbate 80 or polyethylene glycol [5,7,27].

Because the present study is a narrative review, it is limited by a lack of a structured and rigorous methodology for searching, selecting, and integrating evidence, which is a typical prerogative of systematic reviews. As a result, the present study is susceptible to several biases, including selection, publication, and reviewer biases.

Nonetheless, narrative reviews can provide a comprehensive summary of a topic and convey information comprehensively and directly, which is more appropriate for clinicians. In addition, the present study may be the first to compare findings of oral lesions detected in SARS-CoV-2-positive individuals and after the administration of COVID-19 EMA-authorized and WHO Emergency Use Listing-approved vaccines between the pediatric and adult populations and summarize hypothesized pathogenicity mechanisms. Further studies should deepen our knowledge of the macroscopic and microscopic features and underlying mechanisms of oral lesions observed in SARS-CoV-2-positive and COVID-19 pediatric subjects, taking into account subsequent viral variants.

Future research should also investigate oral lesion epidemiology, histopathology, and clinical presentation in relation to age, gender, comorbidities, and associated treatments and in relation to SARS-CoV-2 infection, as well as the type and number of doses of the COVID-19 vaccine administered in the pediatric population.

## 5. Conclusions

The most common oral lesions in pediatric patients positive for SARS-CoV-2 were erosive–ulcerative lesions and macules/petechiae, which were primarily erythematous. The possible pathogenic mechanisms responsible for such oral lesions following SARS-CoV-2 infection include the direct cytopathic effects of the virus on epithelial or endothelial cells, resulting in increased cell permeability and direct cell damage; immune–inflammatory dysregulation, such as an immune–inflammatory hyper-reactions due to COVID-19-induced cytokine storms, especially in MIC-S; host immune–inflammatory hypo-reactions that could promote opportunistic infections; COVID-19 pharmacological therapies that could lead to immunosuppression; or mechanical ventilation causing friction and compression in the mucosa.

In the context of COVID-19 vaccination, oral adverse reactions have been shown to be rare and typically present as erosive–ulcerative lesions with EM-like or unspecified patterns. The potential pathogenic mechanisms responsible for such oral lesions include various hypersensitivity reactions, e.g., those related to molecular mimicry, cross-reactivity, autoimmunity, the reactivation of latent viral infections, and allergies to vaccine adjuvants, such as polysorbate 80 or polyethylene glycol.

Deeper insights into oral lesions detectable in SARS-CoV-2-positive subjects and after COVID-19 vaccination in the pediatric population may increase clinicians’ ability to improve multidisciplinary pediatric oral and general care.

## Data Availability

Data are available on PubMed, Web of Science, Scopus, and Google Scholar databases.
